# DNA methylation signature is prognostic of choroid plexus tumor aggressiveness

**DOI:** 10.1186/s13148-019-0708-z

**Published:** 2019-08-13

**Authors:** Malgorzata Pienkowska, Sanaa Choufani, Andrei L. Turinsky, Tanya Guha, Diana M. Merino, Ana Novokmet, Michael Brudno, Rosanna Weksberg, Adam Shlien, Cynthia Hawkins, Eric Bouffet, Uri Tabori, Richard Gilbertson, Jonathan L. Finlay, Nada Jabado, Christian Thomas, Martin Sill, David Capper, Martin Hasselblatt, David Malkin

**Affiliations:** 1 Genetics and Genome Biology Program, Hospital for Sick Children, PGCRL, 686 Bay Street, Toronto, Ontario M5G 0A4 Canada; 2 Center for Computational Medicine, Hospital for Sick Children, PGCRL, 686 Bay Street, Toronto, Ontario M5G 0A4 Canada; 3 Friends of Cancer Research, 1800 M Street, NW, Suite 1050 South, Washington, DC 20036 USA; 4 Department of Computer Science, University of Toronto, 40 St. George Street, Toronto, Ontario M5S 2E4 Canada; 5 Division of Clinical and Metabolic Genetics, Hospital for Sick Children, 555 University Avenue, Toronto, Ontario M5G 1X8 Canada; 6 Paediatric Laboratory Medicine, Hospital for Sick Children, 555 University Avenue, Toronto, Ontario M5G 1X8 Canada; 7 Division of Hematology/Oncology, Hospital for Sick Children, 555 University Avenue, Toronto, Ontario M5G 1X8 Canada; 8 Department of Pediatrics, Hospital for Sick Children, 555 University Avenue, Toronto, Ontario M5G 1X8 Canada; 9 Department of Medical Biophysics, University of Toronto, Princess Margaret Cancer Research Tower, MaRS Centre, 101 College Street, Toronto, Ontario M5G 1 L7 Canada; 10 Department of Oncology, Cambridge Cancer Center, Robinson Way, Cambridge, CB2 0RE England; 11 Neuro-Oncology Program, Nationwide Children’s Hospital and The Ohio State University, 700 Children’s Dr, Columbus, OH 43205 USA; 12 Division of Hematology/Oncology, Montreal Children’s Hospital of the McGill University Health Centre (RI-MUHC), 1001 Decarie Blvd, Montreal, Québec, H4A 3 J1 Canada; 13 Institute of Neuropathology, University Hospital Münster, 48149 Münster, Germany; 14 Hopp Children’s Cancer Center at the NCT Heidelberg (KiTZ), Im Neuenheimer Feld 280, 69120 Heidelberg, Germany; 15 Division of Pediatric Neurooncology, German Cancer Research Center (DKFZ) and German Cancer Consortium (DKTK), Im Neuenheimer Feld 280, 69120 Heidelberg, Germany; 16 Charité Universitätsmedizin Berlin, corporate member of Freie Universität Berlin, Humboldt-Universität zu Berlin, and Berlin Institute of Health, Department of Neuropathology, Charitéplatz 1, 10117 Berlin, Germany; 17 German Cancer Consortium (DKTK), Partner Site Berlin, Invalidenstrasse 80, 10117, Berlin, German Cancer Research Center (DKFZ), Im Neuenheimer Feld 280, 69120 Heidelberg, Germany

**Keywords:** DNA methylation, Choroid plexus tumors, HumanMethylation450 arrays, Quantitative sodium bisulfite pyrosequencing

## Abstract

**Background:**

Histological grading of choroid plexus tumors (CPTs) remains the best prognostic tool to distinguish between aggressive choroid plexus carcinoma (CPC) and the more benign choroid plexus papilloma (CPP) or atypical choroid plexus papilloma (aCPP); however, these distinctions can be challenging. Standard treatment of CPC is very aggressive and often leads to severe damage to the young child’s brain. Therefore, it is crucial to distinguish between CPC and less aggressive entities (CPP or aCPP) to avoid unnecessary exposure of the young patient to neurotoxic therapy. To better stratify CPTs, we utilized DNA methylation (DNAm) to identify prognostic epigenetic biomarkers for CPCs.

**Methods:**

We obtained DNA methylation profiles of 34 CPTs using the HumanMethylation450 BeadChip from Illumina, and the data was analyzed using the Illumina Genome Studio analysis software. Validation of differentially methylated CpG sites chosen as biomarkers was performed using pyrosequencing analysis on additional 22 CPTs. Sensitivity testing of the CPC DNAm signature was performed on a replication cohort of 61 CPT tumors obtained from Neuropathology, University Hospital Münster, Germany.

**Results:**

Generated genome-wide DNAm profiles of CPTs showed significant differences in DNAm between CPCs and the CPPs or aCPPs. The prediction of clinical outcome could be improved by combining the DNAm profile with the mutational status of *TP53*. CPCs with homozygous *TP53* mutations clustered as a group separate from those carrying a heterozygous *TP53* mutation or CPCs with wild type *TP53* (*TP53-*wt) and showed the worst survival outcome. Specific DNAm signatures for CPCs revealed *AK1*, *PER2*, and *PLSCR4* as potential biomarkers for CPC that can be used to improve molecular stratification for diagnosis and treatment.

**Conclusions:**

We demonstrate that combining specific DNAm signature for CPCs with histological approaches better differentiate aggressive tumors from those that are not life threatening. These findings have important implications for future prognostic risk prediction in clinical disease management.

**Electronic supplementary material:**

The online version of this article (10.1186/s13148-019-0708-z) contains supplementary material, which is available to authorized users.

## Introduction

Choroid plexus tumors (CPTs) are rare neoplasms of the central nervous system. Within this family of tumors, choroid plexus carcinoma (CPC) is a malignant neoplasm, categorized as a grade III tumor by the World Health Organization (WHO). In contrast, choroid plexus papilloma (CPP) is benign and classified as a grade I tumor, while atypical choroid plexus papilloma (aCPP) is a grade II tumor [1]. CPTs account for 0.4–0.6% of all brain tumors; however, in children, CPTs represent 1 to 4% of all childhood brain tumors, with up to 20% occurring during the first year of life [1]. CPCs account for 20–40% of all choroid plexus tumors in children [1].

Distinction between these tumor subtypes for accurate diagnosis is essential but can be challenging. Current diagnosis relies on assessment of histopathological features, i.e., mitotic activity, cellularity, and nuclear pleomorphism as well as the presence of necrosis and blurring of the papillary growth pattern [2]. Although overall long-term survival for CPPs is relatively favorable (85–100%) after surgical resection alone, CPCs are significantly more aggressive, with a greater tendency for recurrence and less than 50% of patients survive even in the context of combined surgery, chemo- and radiation therapy [3–6]. Most of these children are younger than 3 years of age, and the long-term damaging effects of this therapy on growth and the developing brain are of immense concern, highlighting the need for better biologic risk stratification for tumors in these young patients.

Over 50% of CPC tumors carry somatic mutations in the *TP53* tumor suppressor gene, and *TP53* mutant CPCs have been associated with increased genome instability and poor prognosis [7]. Germline *TP53* mutations have also been observed in ~ 50% of children with CPC which is now considered a component tumor of Li–Fraumeni syndrome (LFS) [8]. We have previously reported that CPTs are highly unstable and harbor unique patterns of chromosome-wide gains and losses [9]. In fact, we demonstrated that differences in copy number (CN) and gene expression distinguish CPCs from CPPs and aCPPs. Nevertheless, despite the use of similar treatment protocols for all patients with CPC, clinical outcomes vary, and our previous findings demonstrate that the clinical variability may be driven by molecular heterogeneity of CPCs.

To improve outcome prediction, more accurate molecular distinction among CPTs is needed. The power of DNAm to identify novel, more molecularly defined tumor subtypes has been established and led to improved stratification and specific tailoring of therapy for patients with a wide range of cancers [10–12]. Recently, Thomas et al. identified three clinically distinct subgroups of choroid plexus tumors by array-based DNAm profiling; however, the methylation groups did not entirely recapitulate the three different histologically defined WHO entities [13]. Based on these observations and our previously reported work, we performed a detailed analysis of the genome-wide methylation profile in our cohort of CPTs with the goal of determining a more accurate classification of the CPT subtypes.

In this study, we performed a comprehensive analysis of DNAm in CPTs and discovered a highly sensitive and specific DNAm signature for CPCs which is able to segregate CPC from other CPT tumors as well as other brain tumors. This signature includes *AK1*, *PER2*, and *PLSCR4* as prospective diagnostic biomarkers for CPC and potentially tractable therapeutic targets.

## Methods

### Patients, tissues, and sample preparation

Institutional Research Ethics Board approval was obtained for the study. Clinical data and tumor samples were collected from many sources, including two large pediatric neuro-oncology centers: the Hospital for Sick Children (SickKids), Toronto, Ontario, and the Children’s Hospital of Los Angeles (CHLA), Los Angeles, CA. The other contributing centers were as follows: St. Jude Children’s Research Hospital, Memphis, TN; the Collaborative Human Tissue Network (CHTN) in Columbus, OH; Schneider Children’s Medical Center, Tel-Aviv, Israel; Montreal Children’s Hospital, Montreal, Quebec; and University of Colorado Health Sciences Center, Denver, CO. Informed consent was obtained from the parents/legal guardians of all patients. Pathologic review of CPTs was conducted by Dr. C. Hawkins. In all other institutions, expert neuropathologists critically examined each case. All samples were processed as described in detail in our previous work [9]. DNA was isolated from either fresh snap frozen (*n* = 53) or formalin-fixed paraffin-embedded (FFPE; *n* = 2) or optimal cutting temperature (OCT) compound (*n*=1) tumor samples. Tumor DNA was extracted using standard phenol–chloroform extraction from fresh frozen samples and the RecoverAll Total Nucleic Acid Isolation Kit for FFPE (Ambion) from FFPE samples.

Our primary cohort comprised 34 samples, including 15 CPPs, 5 aCPPs, and 14 CPCs. An additional validation cohort comprised 22 samples, including 4 CPPs, 3aCPPs, and 15 CPCs. We also used a replication cohort of 61 CPTs from Neuropathology, University Hospital Münster, Germany, for sensitivity testing of the CPC-specific DNAm signature [13]. Patient characteristics are provided in Additional file 1: Tables S1 and S2.

### *TP53* gene sequencing

Sequencing of the coding region of *TP53* (exons 2–11 as well as up to 50 bases into spanning introns of the *TP53* gene) was performed in the molecular diagnostic laboratory at The Hospital for Sick Children in Toronto by direct Sanger sequencing of genomic DNA as previously described [7].

### DNA methylation

Genomic DNA (~ 1 μg) from CPT patients (Additional file 1: Table S1) was treated using sodium bisulfite (Qiagen) converting unmethylated cytosine to uracil but leaving methylated cytosine intact. These samples were then hybridized to the HumanMethylation450 BeadChip from Illumina at the Centre for Applied Genomics (TCAG) at the Hospital for Sick Children using the manufacturer’s recommended protocol. Genome-wide DNAm profiles for all 34 primary samples are available through Gene Expression Omnibus (GEO: http://www.ncbi.nlm.nih.gov/geo/), accession number GSE61044.

### Genome-wide DNA methylation analysis

The data generated from the HumanMethylation450 BeadChip arrays were analyzed using the Illumina Genome Studio analysis software and included normalization, quality control measurements, and background correction. We removed probes targeting the X or Y chromosomes, probes near polymorphic sites (targeting CpGs within 5pb of SNPs that have ≥ 1% minor allele frequency in 1000 Genome Project), internal control probes, and non-specific cross-reactive probes [14]. This filtering process resulted in the retention of 414319 CpG methylation sites for further analysis. For each CpG site, the DNAm level was expressed as the average percentage of methylated cytosines, known as the beta value (*β* = mC/(mC + C)). Mapping of CpG sites to regulatory genomic regions was done using the Illumina annotation.

To determine the sites that are differentially methylated between the carcinoma and papilloma groups, we used a non-parametric Mann–Whitney *U* test at each CpG probe. For all subsequent investigations except the biomarker discovery, we applied the statistical significance level at a *p* value < 0.05 (after FDR correction) and an additional cutoff of at least 30% average methylation difference (Δ*β*) between the groups.

For biomarker discovery, more stringent criteria were used, with a *p* value < 0.001 and average methylation difference Δ*β* ≥ 40%. Data were visualized in Qlucore Omics Explorer 3.3 and Partek Genomics Suite 6.6 software using heatmaps and principal component analysis (PCA) plots.

All differentially methylated genes identified in this study were analyzed using Ingenuity Pathway Analysis (IPA) software to identify pathways that are involved in the etiology of the carcinomas.

### Predictive modeling of the CPC-specific signature

Predictive analysis was performed using the Weka machine learning suite (www.cs.waikato.ac.nz/ml/weka) and R package caret [15]. Predictive models were built using CpGs as data attributes. To avoid overfitting, we used options *CfsSubsetEval–BestFirst* to search for the most predictive non-redundant subset of CpGs using the correlation-based feature selection method. For the purposes of predictive analysis, all 34 CPT data samples were labeled as either “carcinoma” (CPC) or “papilloma” (including CPP and aCPP). Leave-one-out (LOO) cross-validation was applied where one sample was withheld; a new epigenetic signature was identified, and a predictive model was built on the remaining 33 samples and then applied to the withheld sample to predict its status as “carcinoma” or “papilloma.” Repeating this process for each of the 34 CPTs gave the measure of the predictive accuracy.

### Multivariate factor analysis

Using 95 CPT samples of the combined datasets of the 34-sample discovery cohort and the 61-sample replication cohort (35 CPC, 33 CPP, 27 aCPP), we performed multivariate factor analysis of the DNAm levels along with other sample genotype and phenotype attributes to identify possible correspondence between them. Beta values in each of the 59 signature CpGs were analyzed jointly with attributes such as age, *TP53* mutation status, recurrence event status, and death event status (Additional file 1: Table S3). See Additional file 3 for details.

### Validation of differentially methylated CpG sites chosen as biomarkers (pyrosequencing analysis)

Three genes were chosen as biomarkers that demonstrate the greatest degree of segregation between CPCs and CPPs, and the corresponding CpG sites were validated for differential methylation between these tumor groups using both the initial discovery panel of 34 samples and a validation set of 22 samples. Quantitative sodium bisulfite pyrosequencing was performed for *AK1* (cg14578146), *PER2* (cg11903188), and *PLSCR4* (cg07038342). All targeted assays were designed using the PyroMark Assay Design Software 1.0 (Qiagen). All primer sets are listed in Additional file 1: Table S4. Sodium bisulfite-modified genomic DNA was amplified using Hot-Start Taq Master Mix (Qiagen) as previously described [16]. Regions of interest were amplified by PCR, and pyrosequencing was carried out using the PyroMark Q24 pyrosequencer (Qiagen) according to the manufacturer’s protocol (Pyro-Gold reagents). Output data were analyzed using PyroMark Q24 1.0.10 Software (Qiagen), which calculates the methylation percentage β for each CpG site, allowing quantitative comparisons.

### Survival analysis

Survival analysis was performed using the Kaplan–Meier method, and curves were compared using both log-rank and Wilcoxon–Gehan chi-square tests. Overall survival (OS) measured time from initial diagnosis to death from any cause or last follow-up.

## Results

### Identification of methylation signature for CPCs and *TP53* mutation groups in CPCs

Analysis of the genome-wide DNAm of primary CPTs did not show significant differences between CPPs and aCPPs (Mann–Whitney *p* value ≥ 0.55 after FDR correction in all 485577 CpG probes) but there was a significant difference in the CpG methylation profile between CPCs and CPPs or aCPPs (Mann–Whitney *p* value ≤ 0.048, FDR corrected in 51479 CpG sites). Although there were no significant differences in genome-wide DNAm between CPPs and aCPPs, there was a CPC-specific signature in comparison to CPP.

A volcano plot of the genome-wide DNAm profile revealed a general shift towards higher DNAm levels (hypermethylation) in the CPCs as compared to the CPPs (Additional file 2: Figure S1).

**Fig. 1:**
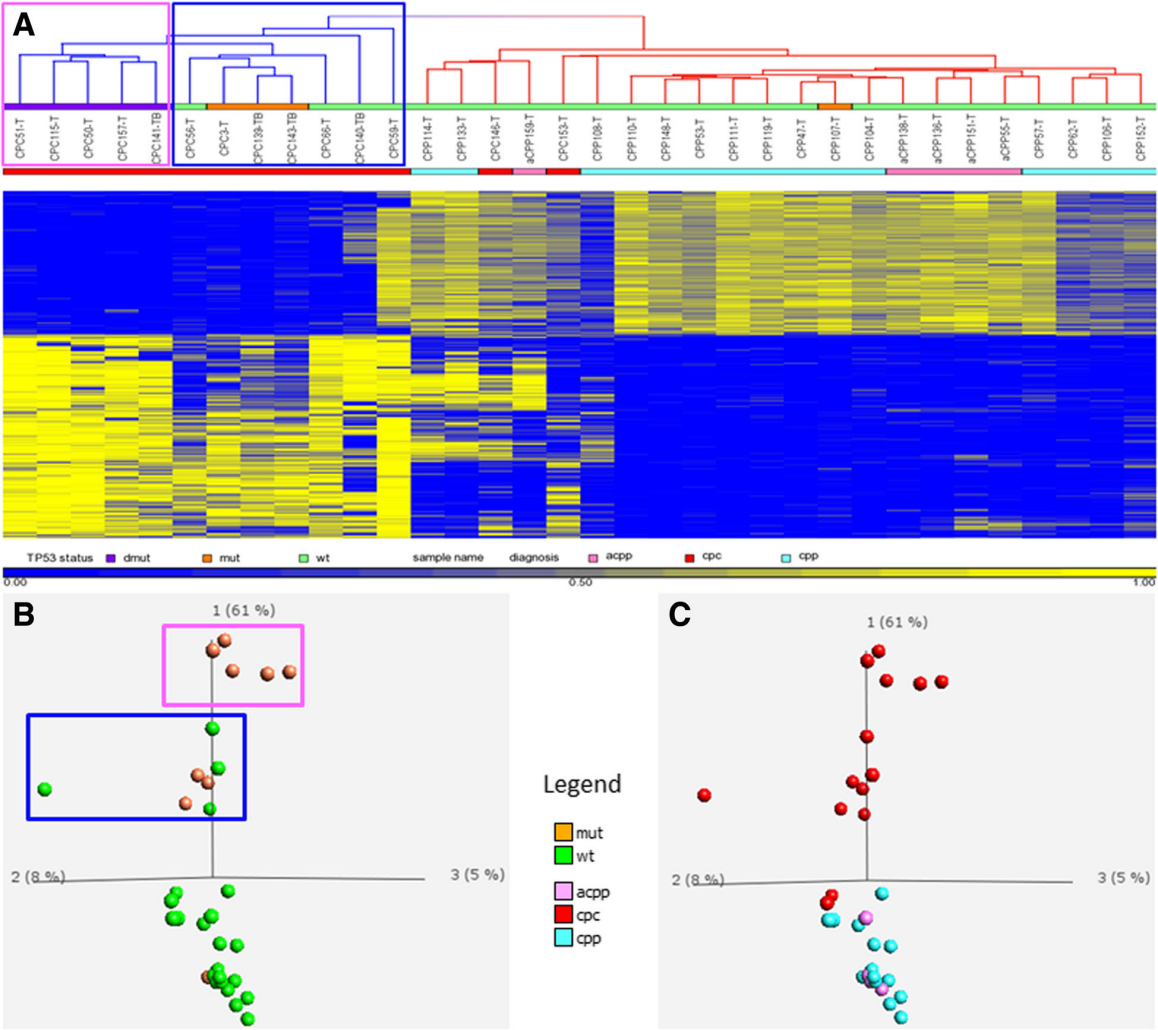
Two-way clustering performed on 34 CPT samples using Pearson’s correlation and average linkage (**a**) and PCA (**b**, **c**) using the top 3361 most differentially methylated CpGs (*p* < 0.05 after FDR correction and at least 30% methylation difference) shows segregation between majority of carcinomas (cpc) and papillomas (cpp and acpp). In addition, we observed segregation within the CPC group based on *TP53* status. Homozygous *TP53*-mut (mutant) = violet bar (**a**) and outlined in pink box (**a**, **b**); heterozygous *TP53*-mut = orange bar (**a**) and outlined in blue box (**a**, **b**); *TP53*-wt (wild type) = green bar (**a**) and green dots (**b**); diagnosis: acpp = pink, cpc = red, and cpp = turquoise (**a**–**c**). The numbers 1, 2, and 3 in PCA plots represent component 1, component 2, and component 3

Using an average beta value difference of 0.3 or greater and an FDR adjusted *p* value < 0.05, we identified a total of 3361 CpG probes that showed significant differences in methylation between CPCs and CPPs. Among these 3361 CpGs, 1388 (or 41%) were hypomethylated and the remaining 1973 CpG sites (or 59%) were hypermethylated in CPCs compared to CPPs. Two-way clustering performed using Pearson’s correlation and average linkage for both the sample tree and the gene tree revealed segregation between the majority (86%) of CPCs and CPPs or aCPPs (Fig. 1).

Since over 50% of CPC tumors carry somatic *TP53* mutations, and *TP53* mutant CPCs have been associated with poor prognosis [7], we sought to determine whether the clustering observed within the CPC group correlated with *TP53* mutation status.

**Fig. 2:**
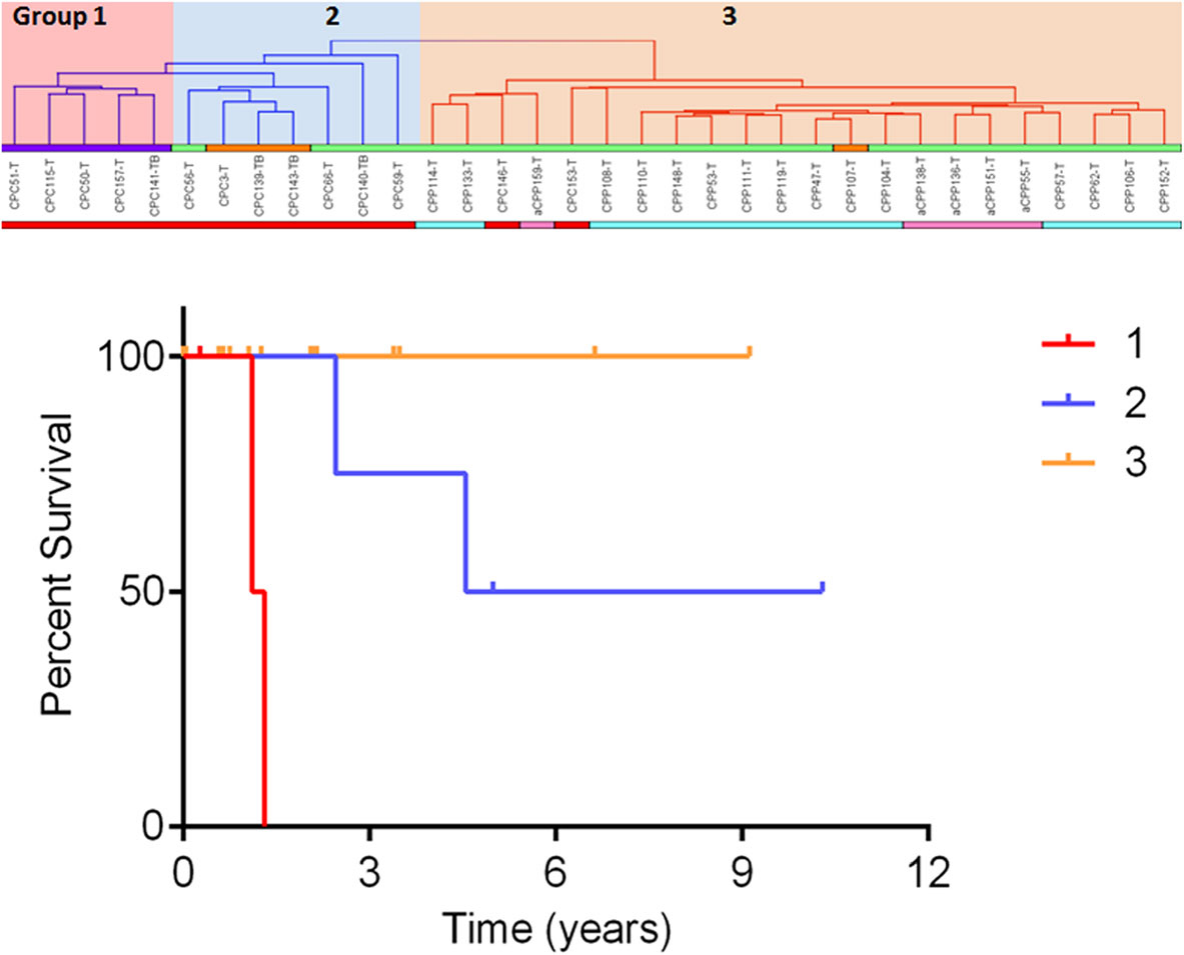
Kaplan–Meier (KM) curve depicting overall survival (OS) estimates of patients with CPT by methylation subgroups. Statistical values comparing the three KM curves were obtained with the log-rank chi-square = 16.7 (df = 2), *p* value 0.0002; and Wilcoxon–Gehan chi-square = 15.5 (df = 2), *p* value 0.00043. Group 1 (pink highlight in top panel, red line in KM plot (*n* = 5)), CPCs with homozygous *TP53-mut* (mutant) = violet bar; group 2 (blue highlight in top panel, blue line in KM plot (*n* = 7)), CPCs with heterozygous *TP53*-mut = orange bar and *TP*53-wt (wild type) = green bar; group 3 (orange highlight in top panel, orange line in KM plot (*n* = 22)), CPPs-heterozygous *TP53-mut* = orange bar and *TP*53-wt = green bar and two CPCs with *TP53-*wt = green bar; diagnosis: cpc = red, acpp = pink, cpp = turquoise

As shown in Fig. 1a, b, CPCs harboring homozygous mutations in *TP53* clustered as a group separate from those carrying a heterozygous *TP53* mutation or CPCs with *TP53-*wt. Survival analysis performed on CPTs (Fig. 2) showed significantly worse outcomes for methylation group 1 and 2, which contained only CPC samples (with mutated *TP53* or *TP53*-wt*)*, than for the methylation group 3 that contained predominantly CPPs or aCPPs and had no death events. Methylation group 1 comprising only CPCs with homozygous *TP53* mutation had a particularly poor prognosis compared to methylation group 2 which contained CPCs with heterozygous *TP53* mutation or the *TP53-*wt (log-rank *χ*^2^ = 16.7 with df = 2, *p* value 0.00023; and Wilcoxon–Gehan *χ*^2^ = 15.5 with df = 2, *p* value 0.00043).

### Pathway and biological function analysis of differentially methylated genes

**Fig. 3:**
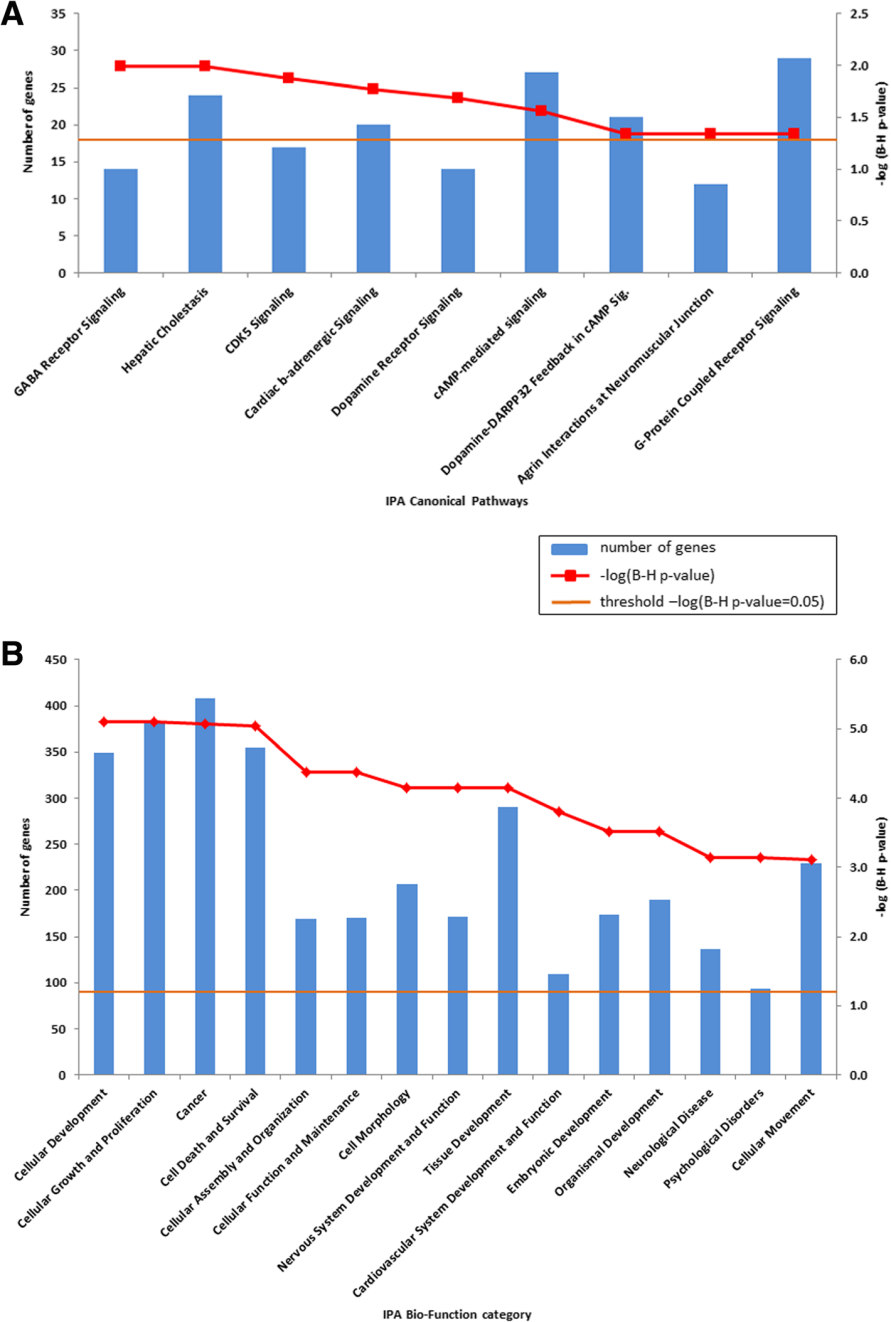
Bar chart shows the enriched canonical pathways (**a**) or enriched biological function categories (**b**) in CPCs using Ingenuity Pathway Analysis (IPA). Major *Y*-axis on the left shows the number of differentially methylated genes. Secondary *Y*-axis on the right shows the significance levels (−log (B-H *p* value 0.05)) of the canonical pathway (**a**) and (−log (B-H *p* value 0.001)) of the biological function category (**b**). The orange line shows the significance threshold cutoff of −log (B-H *p* value 0.05). B-H, Benjamini–Hochberg multiple testing correction

To investigate whether genes affected by alterations of DNAm in CPCs tend to fall within specific molecular pathways, we next performed pathway analysis using Ingenuity Pathway Analysis (IPA) software on a 1328 gene set overlapping the 3361 CpG probes. We identified nine canonical pathways that were significantly enriched in CPCs (FDR corrected *p* value < 0.05, with between 14 and 29 differentially methylated genes in each pathway) in comparison to CPPs (Fig. 3a). As shown in Fig. 3a, the most significantly affected pathway was GABA receptor signaling where 14 genes showed differential methylation between CPCs and CPPs or aCPPs. We observed hypomethylation in the body of *GABA(A)* receptor subunits alpha 4-5 and gamma 3, as well as hypermethylation in the promoter region of subunit gamma 2 in CPCs compared to CPPs (Additional file 1: Table S5).

IPA Biological Function analysis revealed 54 functional categories that were significantly altered (FDR corrected *p* value < 0.05) in CPCs. The most significantly enriched categories in CPCs (with *p* values below 0.001 and including between 94 and 408 differentially methylated genes) were associated with cellular development, cellular growth and proliferation, cancer, cell death and survival, and cellular assembly and organization (Fig. 3b).

### Genomic enrichment of the CPC DNA methylation signature

In order to identify regulatory regions that are enriched in the CPC DNAm signature, we used the Illumina annotation for regulatory regions and compared the 3361 sites with significant differential DNAm in CPCs against the full Illumina 450K array dataset. We identified significant enrichment of CpG sites associated with CPCs in enhancer regions, DNase I hypersensitive sites (DHS) (which are associated with open chromatin and hence active transcription), cancer-specific differentially methylated regions (cDMR), reprogrammed-specific differentially methylated regions (rDMR), and non-CpG island sites. However, we did not find any enrichment at CpG island shores and shelves (Additional file 2: Figure S2).

### Correlation between DNA methylation and gene expression

**Fig. 4:**
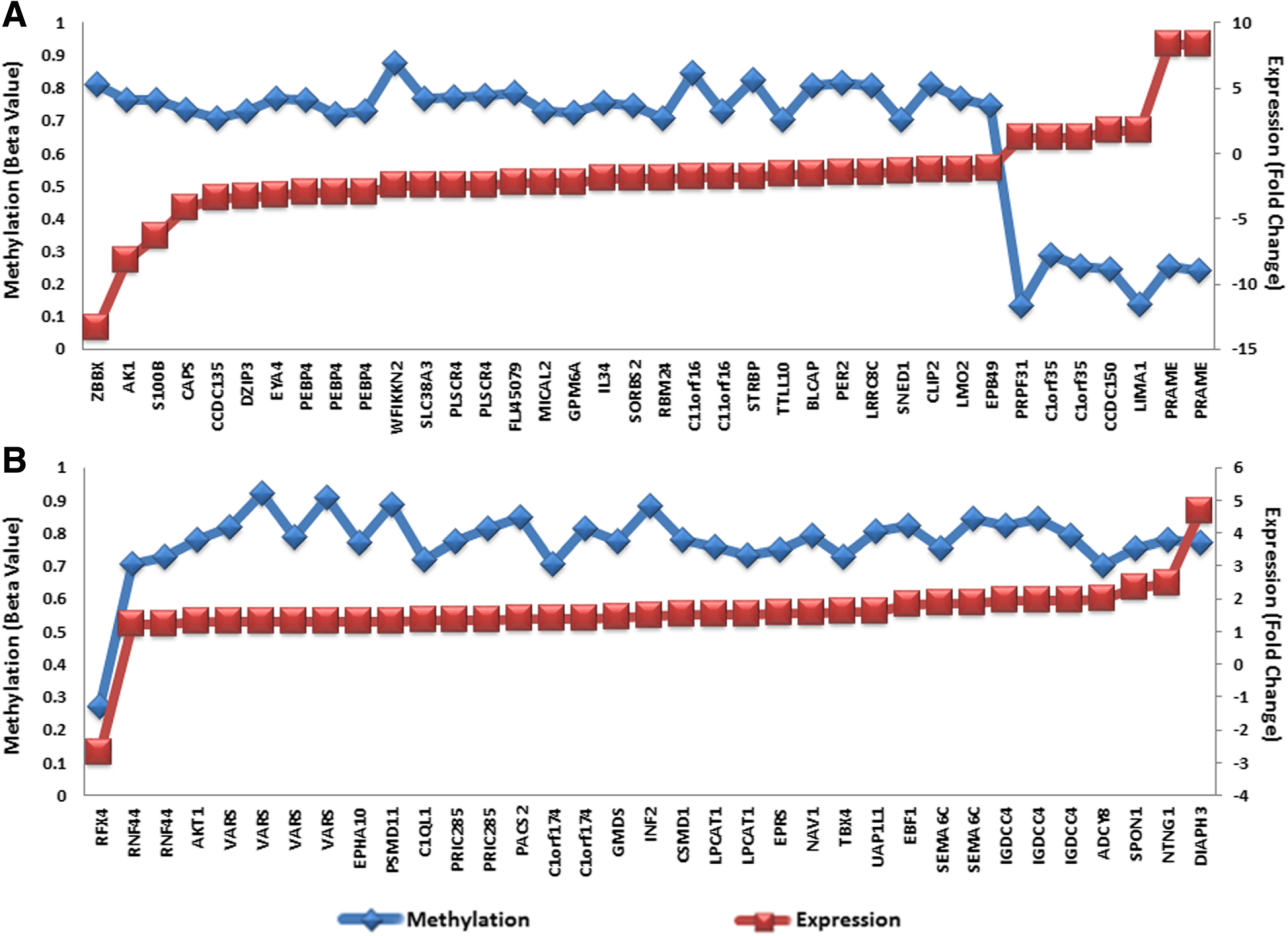
Correlation between promoter (**a**) and body (**b**) methylation and gene expression. Scatter plots showing correlation of 73 CpG sites encompassing 57 single genes between methylation and expression. *Y*-axis on the left of each plot shows methylation levels (AVG_Beta Value) as a mean over all CPC samples (blue), and secondary *Y*-axis on the right shows expression fold changes of a given gene in CPCs vs CPPs (red)

To assess the impact of differential methylation on gene expression, we examined the 3361 CpG sites in a dataset of 40 CPTs analyzed previously using Affymetrix Exon1 microarrays [9]. We identified 26 samples from this study that had expression profiling data available; of those, we had 11 CPCs, 12 CPPs, and 4 aCPPs. Differential expression between CPCs and CPPs was determined at the significance level with FDR corrected *p* value < 0.05. We identified 57 single genes showing correlation between methylation and expression levels in CPCs, suggesting an epigenetic regulation of gene expression for these important genes in CPCs. Namely, 32 genes displayed differential methylation in their promoter region (comprising the region 1500 bp upstream of the transcription start site (TSS), the 5′UTR region, and the first exon), which was inversely correlated with their expression in CPCs (Fig. 4a and Additional file 1: Table S6). Meanwhile, 25 genes demonstrated differential methylation of probes within the gene body, which was positively correlated with their expression (Fig. 4b and Additional file 1: Table S7).

### CPC-specific DNA methylation signature

**Fig. 5:**
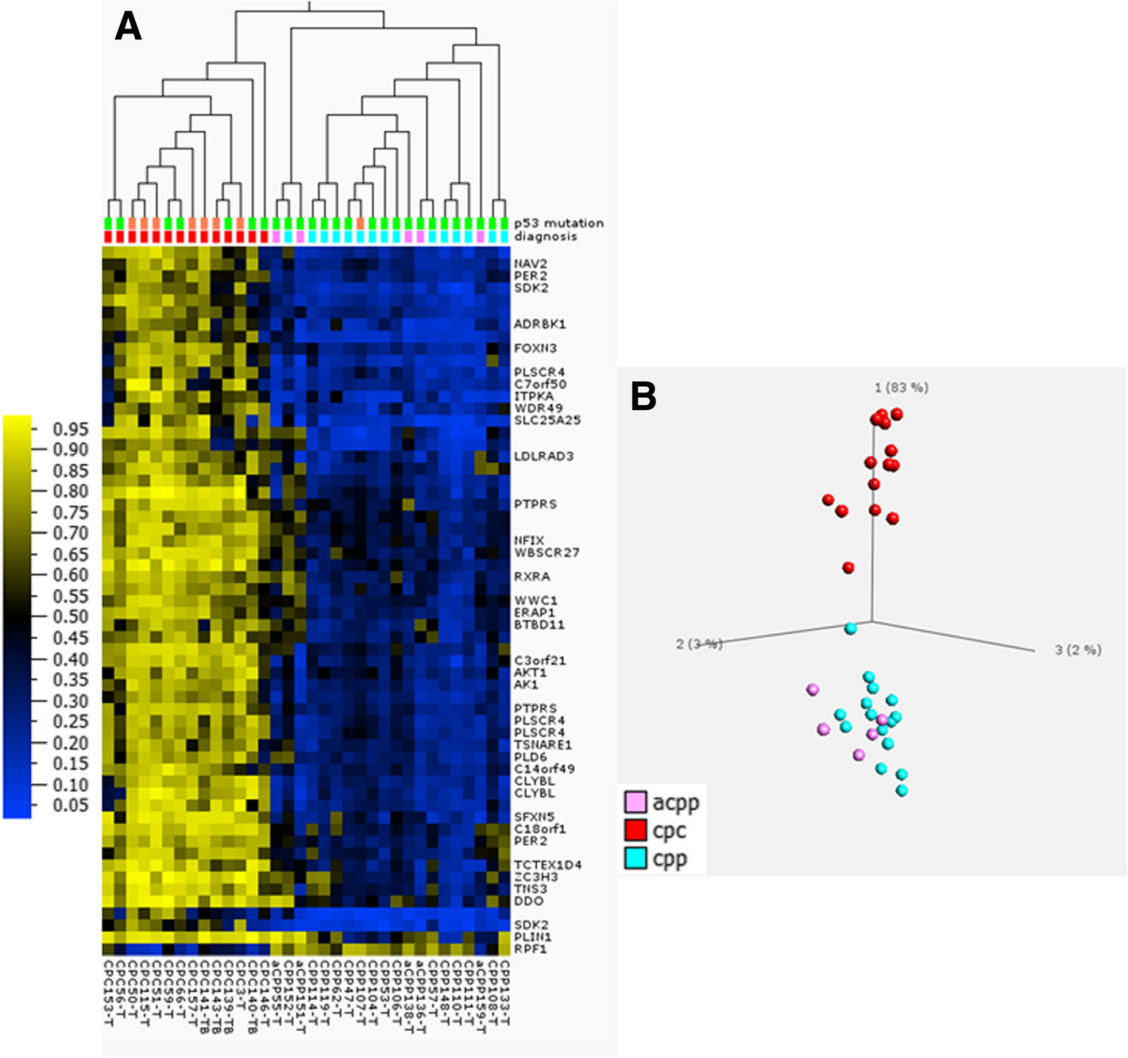
CPC specific DNA methylation signature of 34 CPT samples Heatmap (**a**) and PCA (**b**) of 59 differentially methylated CpG sites encompassing 33 candidate genes extracted from the dataset of 3361 CpG sites by applying increased stringency (*p* < 0.001 and at least 0.4 delta beta). Hierarchical clustering was done using Euclidean metric. High methylation = yellow; low methylation = blue; *TP53* status: wild type = green, mutated = orange; diagnosis: cpc = red, acpp = pink, cpp = turquoise. The numbers 1, 2, and 3 in PCA plot represent component 1, component 2, and component 3.

We further increased the differential methylation stringency (*p* value < 0.001 after FDR correction and Δ*β* > 0.4 by magnitude), which allowed us to extract 59 high-confidence differentially methylated CpG sites encompassing 33 candidate genes (Fig. 5 and Additional file 1: Table S8).

We chose CpG sites representing 3 genes (*AK1—*adenylate kinase, *PER2—*period circadian clock 2, and *PLSCR4—*phospholipid scramblase 4) for the validation of their differentially methylated status by targeted quantitative pyrosequencing. The selection was based on satisfying the following criteria: (1) CpG sites methylated in the promoter region, (2) inverse correlation between methylation and expression, and (3) known biological association with cancer. Results obtained by pyrosequencing using the discovery subset of 34 CPT tumors as well as the validation subset of 22 CPT samples are presented in Additional file 2: Figure S3. For all three genes, bisulfite pyrosequencing validated the findings from the methylation array analysis and confirmed that the methylation at the tested CpG sites was higher in CPCs than in CPPs. In addition, we visualized DNAm profile of the 3 biomarker CpGs, obtained from the initial discovery subset of 34 CPTs using the HumanMethylation450 array and from the validation subset of 22 CPTs using pyrosequencing. We found that these 3 biomarkers were sufficient to segregate CPCs from the CPPs or aCPPs, proving them to be a highly specific minimal CPC DNAm signature (Additional file 2: Figure S4).

Direct comparison of the BeadChip and pyrosequencing values for the 34 cases for which data were available from both methods revealed a high correlation between the two methodologies, with *r*^2^ between 0.90 and 0.99. Correlation plots for each tested CpG are shown in Additional file 2: Figure S5.

### Accuracy measurement of CPC DNA methylation signature

We applied several types of machine learning models to the DNAm profiles of 14 CPC samples and 20 CPP or aCPP samples. Our goal was to predict the CPT status of a sample using the methylation values in signature CpGs as data attributes. Leave-one-out (LOO) cross-validation was used to determine the predictive accuracy.

First, we used only three CpGs as data attributes representing the minimal signature genes: *AK1* (cg14578146), *PER2* (cg11903188), and *PLSCR4* (cg07038342). Even with this minimal data representation, several predictive model types, such as logistic regression, multilayer perceptron neural network, and Naïve Bayes model, were able to predict carcinoma status on all left-out samples with perfect accuracy (area under ROC = 100% in each case). The logistic regression model showed that cg11903188 (gene PER2) was the most predictive of the three, with an odds ratio = 7.33, followed by cg07038342 (*PLSCR4*, odds ratio = 3.03) and cg14578146 (*AK1*, odds ratio = 1.20).

Next, we explored the predictive accuracy and robustness of the full epigenetic signature using LOO cross-validation. A full new epigenetic signature was identified for each of the 34 cross-validation folds using the same parameters (*p* value < 0.001 after FDR correction and Δ*β* > 0.4 by magnitude). This signature was then used to build predictive models of several types, both without feature selection (i.e., using all CpGs as data attributes) and with feature selection to optimize model accuracy by using only the most predictive non-redundant CpGs. We applied several types of machine learning models available through the R package caret, such as logistic regression, support vector machines, and random forests, to detect carcinoma cases. In all such results, we achieved perfect 100% specificity, i.e., no false positives. However, the carcinoma sample CPC146-T was persistently misclassified by all models and occasionally the models also failed to detect another sample CPC153-T, thereby giving a sensitivity estimate between 85.7 and 92.9% (1 or 2 false positives out of 14 CPC samples). These observations match well with the results shown in Fig. 1 where the same two CPC samples are clustered within the papilloma group. Overall, our estimates of a perfect specificity and a high sensitivity suggest a good potential of the CPC-derived DNAm signature in predicting carcinomas.

### Testing the specificity of CPC DNA methylation signature using external datasets

**Fig. 6:**
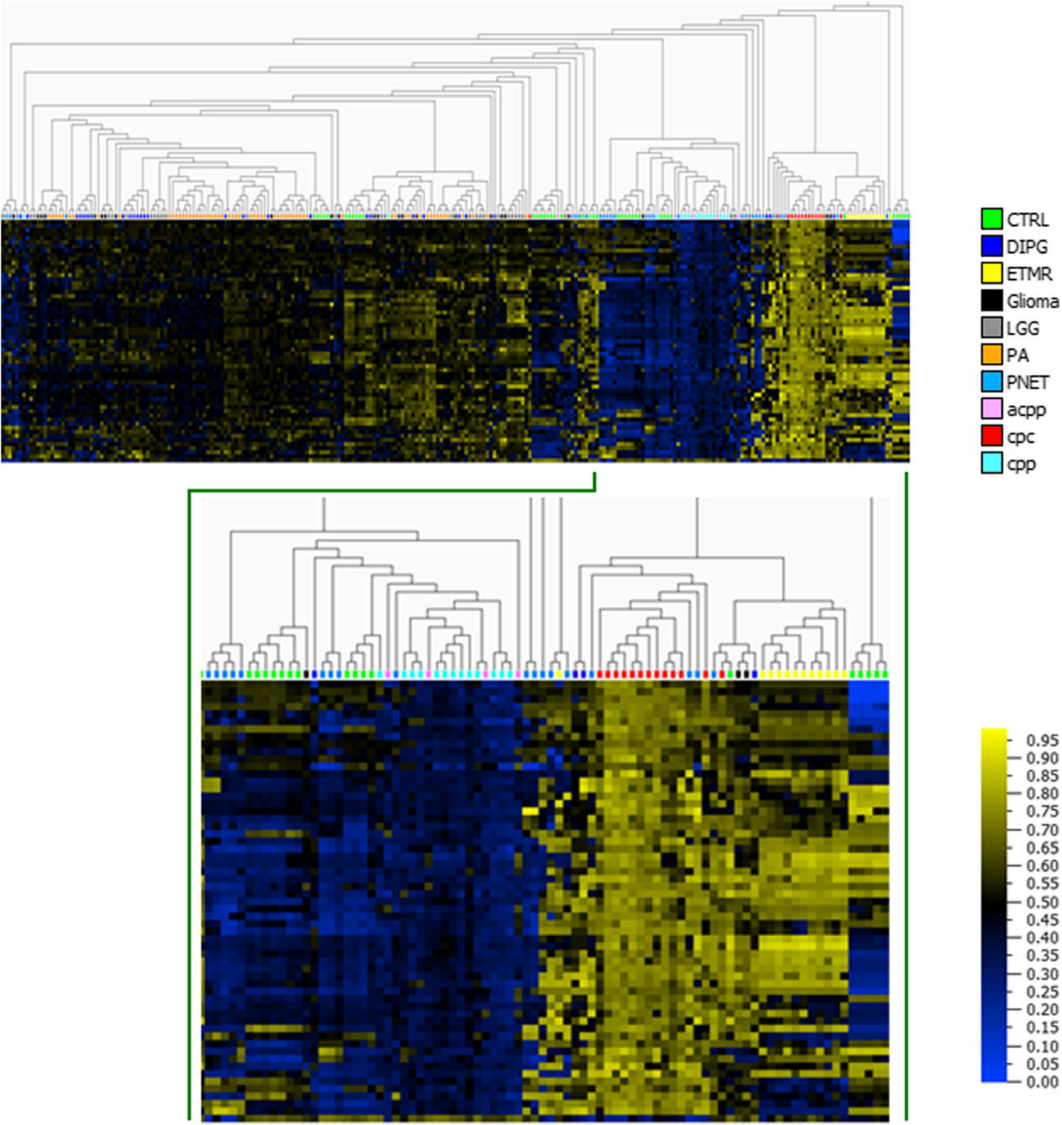
Heatmap of brain tumor DNA methylation datasets from GEO and TCGA databases with CPC-specific DNA methylation signature (59 differentially methylated CpG sites). Comparison of CPC-specific DNA methylation signature to other brain tumors. Brain tumor data were derived from GEO database under accession number GSE50022—for diffuse intrinsic pontine glioma (DIPG) (*n* = 28), GSE44684—from pilocytic astrocytoma (PA) *n* = 61-PA and *n* = 6 normal cerebellum (CTRL)), and GSE52556—from embryonal tumors with multilayered rosettes (ETMR) (*n* = 12), primitive neuroectodermal tumors (PNET) (*n* = 28), normal brain (CTRL) (*n* = 34), and TCGA-glioma (*n* = 24) and low grade glioma (LGG) (*n* = 30) from TCGA. CPTs diagnosis: cpc = red, acpp = pink, cpp = turquoise. Hierarchical clustering was done using Euclidean metric. High methylation = yellow; low methylation = blue

To further validate the specificity of the CPC biomarker signature, we extracted publicly available pediatric brain tumor DNAm datasets from the GEO database as well as from the TCGA (The Cancer Genome Atlas) Data Portal (http://portal.gdc.cancer.gov/) of adult origin and analyzed them jointly with our CPT methylation data. The brain tumor DNAm datasets derived from the Illumina HumanMethylation450 array consisted of 28 diffuse intrinsic pontine gliomas (DIPG) (GSE50022) and 67 pilocytic astrocytomas (GSE44684), 12 embryonal tumors with multilayered rosettes (ETMR) and 28 primitive neuroectodermal tumors (PNET) (GSE52556) from GEO, and 24 glioma and 30 low grade glioma (LGG) from TCGA. The extracted data were combined with our CPT data, all samples were restricted to the CpGs comprising the CPC-specific signature, and hierarchical clustering was used to examine the separation of CPTs from other brain tumors (Fig. 6). The heatmap of hierarchical clustering shows distinct DNAm patterns for CPCs. In addition, CPCs segregate from the majority of the other brain tumors, further confirming the high specificity of the CPC DNAm signature (Fig. 6).

### Testing the sensitivity of the CPC DNA methylation signature using a replication cohort of CPT tumors

**Fig. 7:**
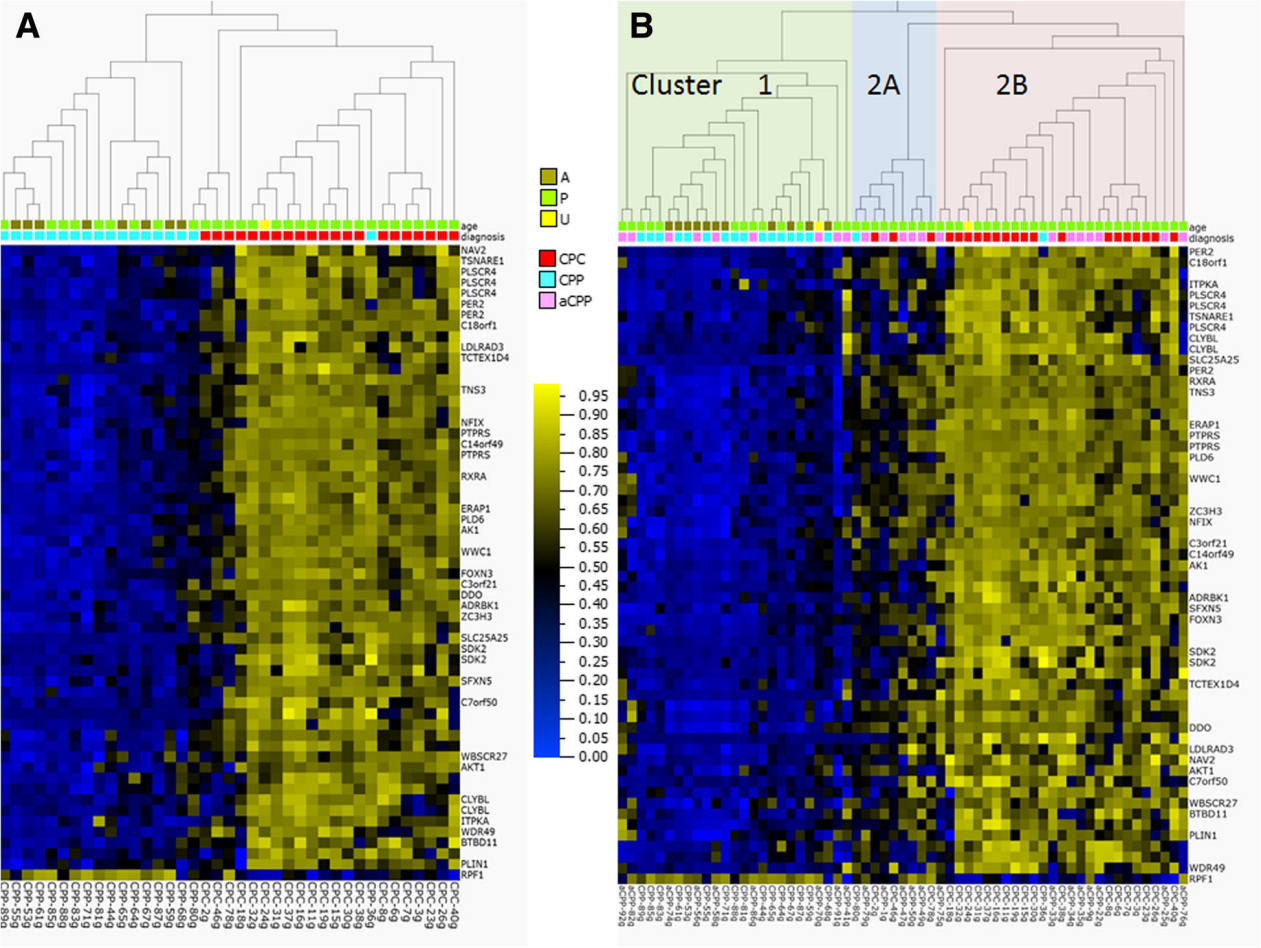
DNA methylation profile derived from the replication dataset of CPTs after applying CPC specific DNA methylation signature (59 differentially methylated CpG sites). **a** DNA methylation profile of 39 CPT samples (18 CPPs and 21 CPCs). **b** DNA methylation profile of 61 CPT samples. Hierarchical clustering was done using Euclidean metric. High methylation = yellow; low methylation = blue; diagnosis: cpc = red; acpp = pink; cpp = turquoise; age: A = adults, P = pediatric cases, U = unknown

We applied the CPC-specific DNAm signature to a cohort of CPTs derived from a recently published study [13]. This cohort consisted of 21CPCs, 22aCPPs, and 18CPPs. Using this replication dataset, CPCs clustered together and separate from CPPs. This indicates high sensitivity for the CPC-specific DNAm signature in properly assigning the histopathological profile to CPTs (Fig. 7a). Only two CPPs (CPP80g and CPP36g) clustered with the CPC; interestingly, the histopathological report for CPP36g showed that this specific CPP has increased cellularity, blurring of the papillary growth pattern, but no mitotic activity, no tumor necrosis, and no nuclear pleomorphism, features not commonly associated with CPP histopathology. This data suggests that the CPC-specific DNAm signature can be more specific in classifying subgroups of CPT tumors based on their methylation profiles compared to histopathological classification.

**Fig. 8:**
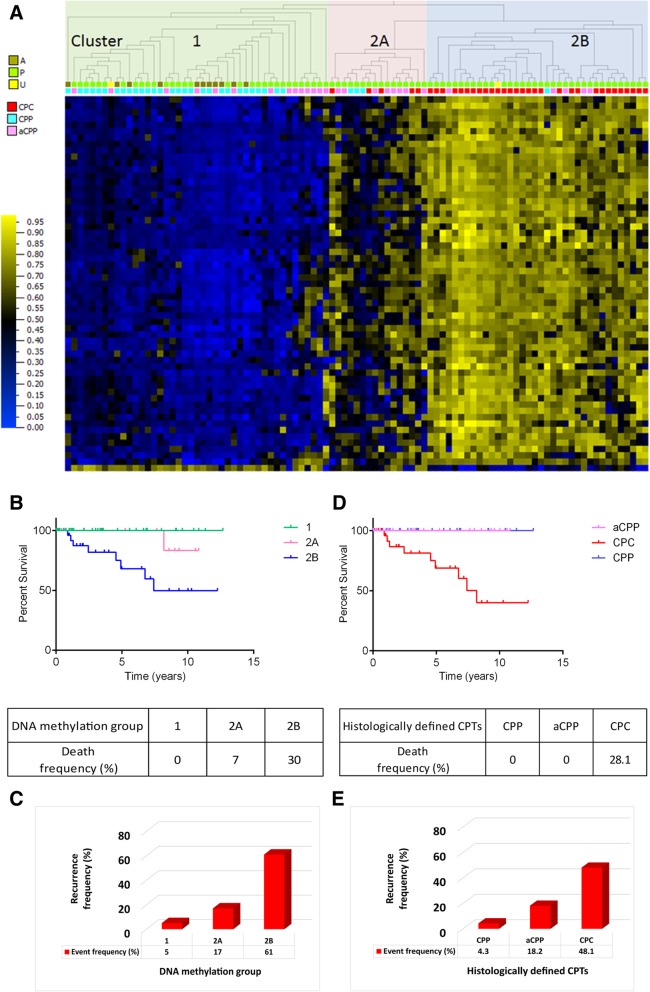
DNA methylation profile derived from the combined discovery and replication datasets of CPTs after applying CPC specific DNA methylation signature (59 differentially methylated CpG sites), Kaplan–Meier plots showing overall survival by methylation subgroups or by histological diagnosis along with tables showing frequency of death event and histograms showing frequency of recurrence events within each of the DNA methylation signature derived clusters as well as for each of the histologically defined CPTs. **a** DNA methylation clusters of the 95 CPT samples (combined datasets of 34 samples used in discovery cohort and 61 samples from replication cohort) defined by applying CPC-specific DNA methylation signature. Hierarchical clustering was done using Euclidean metric. High methylation = yellow; low methylation = blue; diagnosis: cpc = red, acpp = pink, cpp = turquoise; age: A = adults, P = pediatric cases, U = unknown. Kaplan–Meier curves depicting overall survival (OS) estimates of patients with CPT by methylation subgroups (**b**) or by histological diagnosis (**d**). Statistical values were obtained with the log-rank chi-square = 11.8 (df = 1), *p* value 0.0008, when comparing patients grouped by methylation clusters and with the log-rank chi-square = 0.003495 (df = 2), *p* value 0.9529, when comparing patients grouped by diagnosis. Histograms showing frequency of recurrence event in each of the clusters derived from DNA methylation signature (**c**) of 95 CPT samples or for each of the histologically defined CPTs (**e**)

Furthermore, we wanted to check the sensitivity of our CPC-specific DNAm signature in classifying aCPPs from the replication cohort. The first methylation cluster includes most of the CPPs (cluster 1) and some aCPPs as expected. The second cluster is divided into two subclusters (cluster 2A and 2B) where one is enriched in mostly CPCs (2B) and the other one (2A) includes a histologically defined mix of CPCs, CPPs, and aCPPs (Fig. 7b). Hierarchical clustering performed separately for each histologically defined group of CPPs (Additional file 2: Figure S6A) and aCPPs (Additional file 2: Figure S6B) from the replication cohort is able to clearly distinguish a subset within each of the CPP and aCPP groups based on their DNAm profiles, although histologically they were all classified as either CPP or aCPP. Next, we combined samples from two cohorts (34 samples initially used in the discovery of the CPC specific DNAm signature and 61 samples from the replication cohort) to generate a DNAm profile based on the CPC-specific signature (Fig. 8a). Using heatmap combined with hierarchical clustering, the data shows that CPT samples are grouped into two main clusters (cluster1 and 2) but cluster 2 is divided into two subgroups (2A and 2B). The distribution of histologically defined CPCs, CPPs, and aCPPs is similar as described above when only a replication cohort of 61 samples was used (Fig. 7b). Based on the distribution of 95 histologically defined CPC, CPP, and aCPP samples within molecularly defined clusters, we also looked at the frequency of either death or recurrence events. The frequency of a death event (Fig. 8b) is very different in each of the molecularly defined groups. In group 1, the frequency of a death event is 0%, whereas it is 30% in group 2B and only 7% in group 2A. The frequency of recurrence is 5% in group 1, (Fig. 8c) 61% in group 2B, and only 17% in group 2A. Frequency of either event is extremely high in group 2B, extremely low in group 1, and significantly reduced in group 2A. Chances of surviving in group 2A are 4 times higher than in group 2B. When estimating either death or recurrence events assessed on histologically defined CPTs, the death event was 28.1% in CPCs and 0% in CPPs or aCPPs (Fig. 8d) whereas the frequency of recurrence was 12.9% lower than projected based on DNAm clusters (Fig. 8e). DNA methylation-based disease risk assessment was significantly improved compared to the frequency of death event assessed on histologically defined CPTs. Seven percent (CPP = 3, aCPP = 8, CPC = 5) of patients could be classified with more favorable outcome compared to 0% when using histopathological criteria alone (Fig. 8d), while the recurrence frequency measured by current criteria was underestimated (Fig. 8e) when compared to frequency assessment based on specific DNAm signature for CPCs. Interestingly, factor analysis revealed that the CPC-specific DNAm signature forms an independent group of data, quite distinct from the available phenotype, genotype, or clinical attributes (Additional file 2: Figures S7 and S8). See Additional file 3 for details. We tested the methylation profiling classifier [12] (Heidelberg classifier at https://www.molecularneuropathology.org) on all samples from cluster 1, 2A, and 2B in Fig. 8 to compare to the CPC-specific DNAm signature classification. The results are presented in Additional file 1: Table S9. In our cluster 2A, we found 5 CPCs which were classified by the Heidelberg classifier [12] as pediatric B (methylation class closely related to methylation cluster 3 described in Thomas et al. [13]). These CPCs represent a group of patients with much more favorable outcome then predicted by the Heidelberg classifier. We also found 3 CPPs and 2 aCPPs from our cluster 1 being classified as pediatric B. Our classification performed better than the Heidelberg classification given that a subset of the CPCs/CPPs/aCPPs with favorable outcome was mixed among the ones with the severe outcome.

## Discussion

We generated a genome-wide DNAm profile in CPTs to address our hypothesis that epigenetic alterations would be associated with specific phenotypes. We undertook a detailed approach to define the differences in DNAm signature for different groups of CPTs as one of the epigenetic factors responsible for CPC development and progression.

We showed significant genome-wide hypermethylation in CPCs compared to either CPPs or aCPPs, but no difference between CPPs and aCPPs. This could be in part due to the fact that we had a smaller number of aCPP cases. A total of 3361 differentially methylated probes showed segregation between the majority of CPCs and CPPs or aCPPs, thus distinguishing the aggressive form of CPTs, that is, CPCs from benign CPTs (CPPs or aCPPs). Unsupervised hierarchical clustering revealed two main clusters within CPCs based on *TP53* mutation status. CPCs with homozygous *TP53* mutations segregated from CPCs with heterozygous *TP53* mutations or CPCs with *TP53-*wt and showed significantly worse survival outcome compared to CPPs and aCPPs or CPCs with heterozygous *TP53* mutations or *TP53-*wt. This is consistent with our previous observation of the worst outcome for CPC tumors carrying homozygous *TP53* mutations [9]. Some *TP53-*wt CPCs cluster within group 2 (together with heterozygous mutated *TP53*) and in our cohort one of the *TP53-*wt patients did not survive, but two *TP53-*wt CPC patients cluster out of the CPC clusters (1 or 2), with CPPs in cluster 3 showing excellent survival. It is hard to predict, based on *TP53* mutational status alone, which CPC patient with *TP53-*wt would have a better prognosis. By combining DNAm and *TP53* mutational status, we show that we can distinguish between *TP53-*wt cases and predict the outcome.

Pathway analysis using IPA identified nine canonical pathways with GABA receptor at the top of the list that were significantly enriched in CPCs in comparison with CPPs or aCPPs. GABA neurotransmitter in the mammalian central nervous system (CNS) acts at either ionotropic (GABA(A)Rs) or metabotropic (GABA(B)Rs) receptors.

Intriguingly, GABA has also emerged as a tumor signaling molecule in the brain and periphery that controls tumor cell proliferation [17, 18]. Recent studies indicate that GABA and its receptors seem to play regulatory effects in many kinds of cancers [19, 20]. Observations that GABA(A) receptor levels change in different tumors and tumor cell lines raise the possibility that manipulating GABA receptor activity might inhibit tumor growth [21]. The GABA(A) receptor allosteric agonist Nembutal has been shown experimentally to inhibit colon cancer growth and metastasis [22]. GABA also plays a role in synchronizing suprachiasmatic nucleus (SCN) neurons [23], and recent evidence suggesting the presence of rhythmic clock gene expression in rat choroid plexus (CP) points to the possible involvement of CP in SCN circadian information [24]. It is possible that deregulated GABR(A) signaling in CPTs has an effect on the choroidal autonomous clock or on synchronization signals/circuits from the SCN neurons. Since the circuits are critical for regulating physiology and behavior, as well as the integration of metabolic information [25], disturbances in the communication between the body clocks can desynchronize the circadian system, which is believed to contribute to the development of many diseases including cancer [26].

In this study, we demonstrate that DNAm profiling can distinguish aggressive forms of CPTs from benign forms of CPTs. We have generated a CPC-specific DNAm signature which includes several important genes such as *AK1*, *PER2*, and *PLSCR4*. This signature is highly specific when compared to other brain tumors highlighting its potential clinical diagnostic utility. When the CPC-specific signature was applied to a replication cohort of 39 samples (18 CPPs and 21 CPCs), CPCs were clustered together, separate from CPPs, but 2 histologically defined CPPs (CPP80g and CPP36g) clustered with CPCs. Thus, we are able to distinguish two distinct molecular subgroups, one comprised of a mixed population of histopathologically defined samples with a majority of one histologically defined subtype and one homogenous subgroup entirely comprised of CPPs.

Also, checking sensitivity of the CPC-specific DNAm signature in classifying the aCPP in the replication cohort of 61 samples (18 CPPs, 21 CPCs, and 22aCPPs), we found that aCPPs are distributed across different molecular subgroups. These data strongly suggest that our CPC-specific DNAm signature is more accurate in classifying CPT tumors based on their methylation profiles compared to histopathological classification alone and that the WHO classification of the CPT based on CPP, aCPP, and CPC can benefit from the addition of the molecular signature to provide a more accurate diagnosis that could impact patient clinical management.

The CPC-specific DNAm signature we described here comprised several genes including *AK1*, *PER2*, and *PLSCR4*. Methylation of the CpG sites in the promoter regions of these genes was more than 30% higher in CPC than in CPPs or aCPPs. This was correlated with significant downregulation at the mRNA level indicating that the transcription of these genes is epigenetically regulated in CPCs.

Emerging evidence has also revealed tight links between the regulation of cellular metabolism [27] and the molecular clock and that the alteration of circadian rhythms might lead to increased susceptibility to cancer in humans [26, 28]. AK1 deficiency, which we observed in our previous study [9], is known to reduce metabolic signal reception by metabolic sensors, such as K-ATP channels and AMP-activated protein kinase (AMPK), compromising their ability to withstand energetic stress [29, 30]. In the context of a circadian clock, AMPK is essential for maintaining metabolic homeostasis and preventing metabolic disorders [31].

The mechanism of dysregulation of circadian genes in cancers includes epigenetic silencing by promoter methylation. We suggest that loss of circadian homeostasis through circadian disruption may be a novel risk factor in choroid plexus tumorigenesis. Decreased expression through hypermethylation of CpG islands or aberrant acetylation in the promoters of the core circadian genes *Per1*, *Per2*, and *Per3* are reported in a spectrum of human cancers [32]. More studies are needed to test the hypothesis that loss of circadian homeostasis through circadian disruption may be a potential risk factor in choroid plexus tumorigenesis.

In our study, hypermethylation of CpG sites in the promoter region of *PER2* is correlated with significantly lower expression of *PER2* in CPCs compared to CPPs or aCPPs. *PER1* and Cryptochrome-2 (*CRY2)* genes belonging to the core clock family of genes showed significantly lower expression in CPCs as well [9].

*PER1* and *PER2* genes are currently considered to be true tumor suppressor genes, as decreased expression of either (or both) has been reported in several types of human cancers [33–35] and has been shown to be an independent predictor of poor prognosis in patients with gastric cancer [36].

Downregulation of *Per2* is correlated with increased levels of Cyclin D and Cyclin E and accelerated tumor growth in vivo [37] whereas induced overexpression of either *Per1* or *Per2* has been shown to inhibit the growth of cancer cells and increase their apoptotic rate [38]. In our previous study [9], we observed increased levels of *Cyclin A2* as well as *Cyclin E1* and *E2* in CPCs compared to CPPs or aCPPs further confirming a correlation between downregulation of *PER2* and increased levels of *Cyclin A* and *Cyclin E* as one of the factors involved in CPCs tumorigenesis.

The findings generated from this study provide a framework for improved molecular stratification for diagnosis and treatment as well as the development of potential prognostic markers to better differentiate aggressive CPT tumors from those that are not life threatening. As well, some of the markers might be predictors of response to particular chemotherapeutic agents as previously reported [39].

Furthermore, our study confirms the relationship between the circadian clock, cancer, and DNAm at clock genes and suggests that improper DNAm may alter clock gene expression, contributing to the etiology of CPCs. The distinct proliferation rhythm between tumor cells and normal cells [40] provides an intriguing opportunity to maximize the effect of anticancer therapies in CPC based on this circadian clock.

In conclusion, we discovered a highly sensitive and specific DNAm signature for CPCs which is able to segregate CPC not only from other CPTs but also from other brain tumors. However, the study is not able to differentiate the proportion of the non-tumor cells. We demonstrate that heterogeneity in the clinical outcome of CPT patients cannot be predicted by histopathological assessment alone, but implementation of the specific DNAm signature for CPCs in association with the morphology of the CPT tumor can significantly improve diagnosis. This signature needs to be validated with larger sample numbers before being used in the clinical setting; however, it shows its usefulness in identifying cases which would otherwise be undertreated or perhaps some which would be overtreated. The intention of making this signature accessible to the scientific–medical community raises awareness of the existence of this potentially more accurate classification.

Incorporation of *CPC-specific DNAm signature* into existing survival prediction based solely on histopathological criteria can significantly improve the estimation of disease outcome. Seven percent of patients could be classified as lower risk compared to 0% when using histopathological criteria alone, while the recurrence frequency measured by current criteria was underestimated when compared to frequency assessment based on specific DNAm signature for CPCs.

DNA methylation profiling enables the subclassification of CPTs into 3 disease subgroups in routinely collected material, and the integration of CPC-specific DNAm signature can significantly improve prognostic risk prediction allowing for informed treatment decision, protecting some young patients from devastating and permanent neurological impairment due to aggressive treatment.

## Additional files

Additional file 1: **Table S1.** Patient information for samples used in DNA methylation experiment. **Table S2.** Patient information for samples in replication cohort used in sensitivity testing of the CPC DNA methylation signature. **Table S3.** Multivariate factor analysis. **Table S4.** Primer sets for quantitative sodium bisulfite pyrosequencing. **Table S5.** GABA receptor DNA methylation status in CPTs. **Table S6.** Differentially methylated genes in cpc vs cpp showing differential expression and reverse correlation with DNA methylation in the promoter region of the gene. **Table S7.** Differentially methylated genes in cpc vs cpp showing differential expression and positive correlation with DNA methylation in the body of the gene. **Table S8.** 39 variables (encompassing 33 single genes) segregating CPCs and CPPs. **Table S9.** Comparison between CPC-specific DNAm signature classification and the Heidelberg classifier. (XLSX 108 kb)

Additional file 2: **Figure S1.** Volcano plot showing significantly differentially methylated CpGs (yellow and blue) between CPCs (carcinomas) and CPPs (papilomas). The *X*-axis shows the difference between average DNAm levels in carcinomas and in papilomas, whereas the *Y*-axis shows the significance as the Mann–Whitney *U p* value (on the logarithmic scale). Each point represents a CpG position from the Illumina HumanMethylation450 BeadChip. CPC tumors show an overall predominance of hypermethylation (yellow) compared with CPPs across the signature CpGs, which were identified using the significance level *p* < 0.05 (or log10(*p*) > 1.30) and DNA methylation difference of at least 30%. **Figure S2.** Functional genomic distribution of CpG sites in CPCs. Genomic enrichment of the CPC DNA methylation signature is presented as percentage of all CpG sites on HumanMethylation450 BeadChip from Illumina (green) or of CpG sites derived from differential analysis using corrected Mann–Whitney *U* *p* value < 0.05 and at least 30% difference in average beta between CPCs and CPPs (orange). **Figure S3.** BoxPlot showing differential methylation of tested CpG sites between CPCs and CPPs or aCPPs. (A) Ak1-cg14578146, (B) PER2-cg11903188, (C) PLSCR4-cg07038342; the number of samples in discovery set was 34 and in validation set 22; *Y*-axis shows average beta values and *X*-axis tumor type: CPC = red, aCPP = pink, and CPP = turquoise. *p* value was generated using two group comparison (t test) and represents significance of the difference in methylation between CPCs and CPPs or aCPPs. ***p < 0.001. **Figure S4.** CPC-specific minimal DNA methylation signature. Heatmap (A) and PCA (B and C) of 3 differentially methylated CpG sites encompassing 3 candidate genes from the dataset of 59 CpG sites of CPC-specific signature. This minimal signature shows segregation between CPCs and CPPs or aCPPs. Hierarchical clustering was done using Euclidean metric. High methylation = yellow, low methylation = blue, discovery set (Illumina HumanMethylation450 BeadChip) on 34 discovery samples = orange, validation set (targeted quantitative sodium bisulfite pyrosequencing) on 34 discovery, and 22 validation samples = green; diagnosis: cpc = red, acpp = pink, and cpp = turquoise. The numbers 1, 2, and 3 in PCA plots represent component 1, component 2, and component 3. **Figure S5.** Correlation plot of DNA methylation values (%) in 34 DNA samples for each tested CpG obtained using the Illumina HumanMethylation450 BeadChip and pyrosequencing. High correlation between the two methodologies was observed with an *r*^*2*^ value of ≥ 0.9; *r*^*2*^—Pearson’s correlation coefficient. **Figure S6.** Hierarchical clustering of DNA methylation profile performed separately for each histologically defined group of 18 CPPs (6A) and 22 aCPPs (6B) from replication cohort. Hierarchical clustering was done using Euclidean metric. High methylation = yellow, low methylation = blue; diagnosis: cpp = turquoise and acpp = pink. **Figure S7.** Factor analysis of DNAm signature along with phenotype and genotype sample attributes. The heatmap shows the magnitude of factor loadings for *n* = 12 factors in each of the data attribute. The DNAm beta values at 59 CpG sites contribute strongly to factors 1–3 and 6–12, for which no other available data attributes contribute. Factor 4 is associated with recurrence status, death status, and P53 mutation status, and factor 5 with age. Several CpGs also show an association with these phenotype attributes, but generally less so that with the DNAm-related factors 1–3. **Figure S8.** Factor analysis of DNAm signature along with phenotype and genotype sample attributes, with recurrence attribute removed. The heatmap shows the magnitude of factor loadings for *n* = 12 factors in each of the data attribute. Factor 4 is associated with age, and factor 6 with death status and P53 mutation status. The DNAm beta values at 59 CpG sites contribute strongly to the rest of the factors, with the strongest association being in the top three factors. (DOCX 15 kb)

Additional file 3: Multivariate factor analysis. (DOCX 16 kb)

## Data Availability

Genome-wide DNAm profiles for all 34 primary samples are available through Gene Expression Omnibus (GEO: http://www.ncbi.nlm.nih.gov/geo/), accession number GSE61044.

## Abbreviations

aCPP: Atypical choroid plexus papilloma
AK1: Adenylate kinase
AMPK: AMP-activated protein kinase
cDMR: Cancer-specific differentially methylated regions
CP: Choroid plexus
CPC: Choroid plexus carcinoma
CpG: Cytosine-phosphate-guanine
CPP: Choroid plexus papilloma
CPTs: Choroid plexus tumors
CRY2: Cryptochrome-2
DIPG: Intrinsic pontine gliomas
DNA: Deoxyribonucleic acid
DNAm: DNA methylation
FFPE: Formalin-fixed paraffin-embedded
GEO: Gene Expression Omnibus
IPA: Ingenuity Pathway Analysis
LFS: Li–Fraumeni syndrome
LGG: Low grade glioma
LOO: Leave-one-out
OCT: Optimal cutting temperature
OS: Overall survival
PCA: Principal component analysis
PER2: Period circadian clock 2
PLSCR4: Phospholipid scramblase
PNET: Primitive neuroectodermal tumors
rDMR: Reprogrammed-specific differentially methylated regions
TCAG: Centre for Applied Genomics
TCGA: The Cancer Genome Atlas
TP53-wt: Wild type TP53
TSS: Transcription start site
WHO: World Health Organization
