# Correction to: DNA methylation signature is prognostic of choroid plexus tumor aggressiveness

**DOI:** 10.1186/s13148-019-0737-7

**Published:** 2019-10-21

**Authors:** Malgorzata Pienkowska, Sanaa Choufani, Andrei L. Turinsky, Tanya Guha, Diana M. Merino, Ana Novokmet, Michael Brudno, Rosanna Weksberg, Adam Shlien, Cynthia Hawkins, Eric Bouffet, Uri Tabori, Richard J. Gilbertson, Jonathan L. Finlay, Nada Jabado, Christian Thomas, Martin Sill, David Capper, Martin Hasselblatt, David Malkin

**Affiliations:** 1 Genetics and Genome Biology Program, Hospital for Sick Children, PGCRL, 686 Bay Street, Toronto, Ontario M5G 0A4 Canada; 2 Center for Computational Medicine, Hospital for Sick Children, PGCRL, 686 Bay Street, Toronto, Ontario M5G 0A4 Canada; 3 Friends of Cancer Research, 1800 M Street, NW, Suite 1050 South, Washington, DC 20036 USA; 4 Department of Computer Science, University of Toronto, 40 St. George Street, Toronto, Ontario M5S 2E4 Canada; 5 Division of Clinical and Metabolic Genetics, Hospital for Sick Children, 555 University Avenue, Toronto, Ontario M5G 1X8 Canada; 6 Paediatric Laboratory Medicine, Hospital for Sick Children, 555 University Avenue, Toronto, Ontario M5G 1X8 Canada; 7 Division of Hematology/Oncology, Hospital for Sick Children, 555 University Avenue, Toronto, Ontario M5G 1X8 Canada; 8 Department of Pediatrics, Hospital for Sick Children, 555 University Avenue, Toronto, Ontario M5G 1X8 Canada; 9 Department of Medical Biophysics, University of Toronto, Princess Margaret Cancer Research Tower, MaRS Centre, 101 College Street, Toronto, Ontario M5G 1 L7 Canada; 10 Department of Oncology, Cambridge Cancer Center, Robinson Way, Cambridge, CB2 0RE England; 11 Neuro-Oncology Program, Nationwide Children’s Hospital and The Ohio State University, 700 Children’s Dr, Columbus, OH 43205 USA; 12 Division of Hematology/Oncology, Montreal Children’s Hospital of the McGill University Health Centre (RI-MUHC), 1001, Decarie Blvd, Montreal, Québec H4A 3 J1 Canada; 13 Institute of Neuropathology, University Hospital Münster, 48149 Münster, Germany; 14 Hopp Children’s Cancer Center at the NCT Heidelberg (KiTZ), Im Neuenheimer Feld 280, 69120 Heidelberg, Germany; 15 Division of Pediatric Neurooncology, German Cancer Research Center (DKFZ) and German Cancer Consortium (DKTK), Im Neuenheimer Feld 280, 69120 Heidelberg, Germany; 16 Department of Neuropathology, Charité Universitätsmedizin Berlin, corporate member of Freie Universität Berlin, Humboldt-Universität zu Berlin, and Berlin Institute of Health, Charitéplatz 1, 10117 Berlin, Germany; 17 German Cancer Consortium (DKTK), Partner Site Berlin, Invalidenstrasse 80, 10117, Berlin, German Cancer Research Center (DKFZ), Im Neuenheimer Feld 280, 69120 Heidelberg, Germany

**Correction to: Clin Epigenetics**

**https://doi.org/10.1186/s13148-019-0708-z**

After publication of the original article [1], authors have requested to add a ‘J’ as middle name for Richard Gilbertson. Hence, full name should be Richard J Gilbertson.

The original article has been corrected.

The publisher apologies for the inconvenience.
