# Supplementary material for: DNA methylation signature is prognostic of choroid plexus tumor aggressiveness
Source: Clin Epigenetics. 2019 Aug 13;11:117. doi: 10.1186/s13148-019-0708-z (PMC6692938; doi:10.1186/s13148-019-0708-z)
Supplement: Supplementary file 2 — Figure S1. Volcano plot showing significantly differentially methylated CpGs (yellow and blue) between CPCs (carcinomas) and CPPs (papilomas). The X-axis shows the difference between average DNAm levels in carcinomas and in papilomas, whereas the Y-axis shows the significance as the Mann–Whitney U p value (on the logarithmic scale). Each point represents a CpG position from the Illumina HumanMethylation450 BeadChip. CPC tumors show an overall predominance of hypermethylation (yellow) compared with CPPs across the signature CpGs, which were identified using the significance level p < 0.05 (or log10(p) > 1.30) and DNA methylation difference of at least 30%. Figure S2. Functional genomic distribution of CpG sites in CPCs. Genomic enrichment of the CPC DNA methylation signature is presented as percentage of all CpG sites on HumanMethylation450 BeadChip from Illumina (green) or of CpG sites derived from differential analysis using corrected Mann–Whitney U p value < 0.05 and at least 30% difference in average beta between CPCs and CPPs (orange). Figure S3. BoxPlot showing differential methylation of tested CpG sites between CPCs and CPPs or aCPPs. (A) Ak1-cg14578146, (B) PER2-cg11903188, (C) PLSCR4-cg07038342; the number of samples in discovery set was 34 and in validation set 22; Y-axis shows average beta values and X-axis tumor type: CPC = red, aCPP = pink, and CPP = turquoise. p value was generated using two group comparison (t test) and represents significance of the difference in methylation between CPCs and CPPs or aCPPs. ***p < 0.001. Figure S4. CPC-specific minimal DNA methylation signature. Heatmap (A) and PCA (B and C) of 3 differentially methylated CpG sites encompassing 3 candidate genes from the dataset of 59 CpG sites of CPC-specific signature. This minimal signature shows segregation between CPCs and CPPs or aCPPs. Hierarchical clustering was done using Euclidean metric. High methylation = yellow, low methylation = blue, discovery set (Illumina HumanMethyla [file 13148_2019_708_MOESM2_ESM.docx]

**Title: DNA methylation signature is prognostic of choroid plexus tumor aggressiveness.**

Malgorzata Pienkowska^1^, Sanaa Choufani^1^, Andrei L. Turinsky^1,2^, Tanya Guha^1^, Diana M. Merino^3^, Ana Novokmet^1*^, Michael Brudno^1,2,4^, Rosanna Weksberg^1,5,8^, Adam Shlien^1,6^, Cynthia Hawkins^1,6^, Eric Bouffet^7,8^ , Uri Tabori^1,7,8^ , Richard Gilbertson^10^ , Jonathan L. Finlay^11^, Nada Jabado^12^, Christian Thomas^13^, Martin Sill^14,15^, David Capper^16,17^, Martin Hasselblatt^13^, David Malkin^1,7,8,9^

**Additional file 2: Figure S1**: Volcano plot showing significantly differentially methylated CpGs (yellow and blue) between CPCs (carcinomas) and CPPs (papilomas). The X-axis shows the difference between average DNAm levels in carcinomas and in papilomas, whereas the Y-axis shows the significance as the Mann-Whitney U p-value (on the logarithmic scale). Each point represents a CpG position from the Illumina HumanMethylation450 BeadChip. CPC tumors show an overall predominance of hypermethylation (yellow) compared with CPPs across the signature CpGs, which were identified using the significance level p < 0.05 (or log10(p) > 1.30) and DNA methylation difference of at least 30%. (TIF)

**Additional file 2: Figure S2**: Functional genomic distribution of CpG sites in CPCs. Genomic enrichment of the CPC DNA methylation signature is presented as percentage of all CpG sites on HumanMethylation450 BeadChip from Illumina (green) or of CpG sites derived from differential analysis using corrected Mann-Whitney U p-value < 0.05 and at least 30% difference in average beta between CPCs and CPPs (orange). (TIF)

**Additional file 2: Figure S3**: BoxPlot showing differential methylation of tested CpG sites between CPCs and CPPs or aCPPs. (A) Ak1-cg14578146, (B) PER2-cg11903188, (C) PLSCR4-cg07038342; The number of samples in discovery set was 34 and in validation set 22; Y axis shows average beta values and X axis tumor type: CPC = red; aCPP = pink; CPP = turquoise. P-value was generated using two group comparison (t-test) and represents significance of the difference in methylation between CPCs and CPPs or aCPPs**. *****: p < 0.001. (TIF)

**Additional file 2: Figure S4**: CPC specific minimal DNA methylation signature. Heatmap (A) and PCA (B&C) of 3 differentially methylated CpG sites encompassing 3 candidate genes from the dataset of 59 CpG sites of CPC specific signature. This minimal signature shows segregation between CPCs and CPPs or aCPPs. Hierarchical clustering was done using Euclidean metric. High methylation = yellow; low methylation = blue; discovery set (Illumina HumanMethylation450 BeadChip) on 34 discovery samples = orange; validation set (targeted quantitative sodium bisulfite pyrosequencing) on 34 discovery and 22 validation samples = green; diagnosis: cpc = red; acpp = pink; cpp = turquoise. The numbers 1, 2 and 3 in PCA plots represent component 1, component 2, and component 3. (TIF)

Additional file 2: Figure S5: Correlation plot of DNA methylation values (%) in 34 DNA samples for each tested CpG obtained using the Illumina HumanMethylation450 BeadChip and pyrosequencing. High correlation between the two methodologies was observed with an *r^2^* value of ≥ 0.9; *r^2^* - Pearson's correlation coefficient. (TIF)

**Additional file 2: Figure S6**: Hierarchical clustering of DNA methylation profile performed separately for each histologically defined group of 18 CPPs (6A) and 22 aCPPs (6B) from replication cohort. Hierarchical clustering was done using Euclidean metric. High methylation = yellow; low methylation = blue; diagnosis: cpp = turquoise; acpp = pink. (TIF)

**Additional file 2: Figure S7**: Factor analysis of DNAm signature along with phenotype and genotype sample attributes. The heatmap shows the magnitude of factor loadings for n=12 factors in each of the data attribute. The DNAm beta-values at 59 CpG sites contribute strongly to Factors 1-3 and 6-12, for which no other available data attributes contribute. Factor 4 is associated with recurrence status, death status and P53 mutation status, and Factor 5 with age. Several CpGs also show an association with these phenotype attributes, but generally less so that with the DNAm-related Factors 1-3. (TIF)

**Additional file 2: Figure S8**: Factor analysis of DNAm signature along with phenotype and genotype sample attributes, with recurrence attribute removed. The heatmap shows the magnitude of factor loadings for n=12 factors in each of the data attribute. Factor 4 is associated with age, and Factor 6 with death status and P53 mutation status. The DNAm beta-values at 59 CpG sites contribute strongly to the rest of the factors, with the strongest association being in the top three factors. (TIF)
