# Supplementary material for: DNA methylation signature is prognostic of choroid plexus tumor aggressiveness
Source: Clin Epigenetics. 2019 Aug 13;11:117. doi: 10.1186/s13148-019-0708-z (PMC6692938; doi:10.1186/s13148-019-0708-z)
Supplement: Supplementary file 3 — Multivariate factor analysis. (DOCX 16 kb) [file 13148_2019_708_MOESM3_ESM.docx]

**Multi-variate Factor Analysis**

Using 95 CPT samples of the combined datasets of the 34 sample discovery cohort and the 61 sample replication cohort (35 CPC, 33 CPP, 27 aCPP ) we performed multi-variate factor analysis of the DNAm levels along with other sample genotype and phenotype attributes to identify possible correspondence between them. Beta values in each of the 59 signature CpGs were analyzed jointly with attributes such as age, P53 mutation status, recurrence event status and death event status (Additional file 1: Table S3). Some of the attribute values were unavailable: P53 status in 9 samples, death status in 8 samples, and recurrence status in 23 samples. The age groups (pediatric or adult) was unknown for 2 individuals, and the exact age in years was unavailable for a total of 9 individuals, although 7 of them were known to be in the pediatric age group. Samples with missing values in these attributes were excluded. There were also 5 instances of missing DNAm values in the dataset, which were imputed with average beta values from the k=11 nearest neighbor samples. Factor analysis was performed in R programming environment using the *factanal* function.

Using all available attributes, there remained 65 samples with no missing values (27 CPC, 18 CPP, 20 aCPP). Analysis revealed that the top three factors were determined exclusively by the DNAm patterns: Factors 1 and 2 were strongly associated with the overall DNAm signature involving most CpGs and several additional CpGs also contributed to Factor 3. No other sample attributes had any significant association with these three factors. Factor 4 showed the strongest association with the recurrence event status but also accounted for P53 mutation status and death status. Factor 5 was strongly associated with age. All subsequent factors represented minor DNAm variations in specific CpG. Increasing the number of factors did not change this pattern. Additional file 2: Figure S7 shows the results for n=12 factors, which capture 85.8% of variation in the dataset. Interestingly, approximately 1/3 of signature CpGs showed a noticeable association (loadings > 0.3) with Factor 4 related to recurrence, P53 mutation and death; and 3 CpGs with Factor 5 related to age. However, for all but one of these CpGs their strongest associations (i.e. largest factor loadings) were with factors related to the DNAm signature pattern. Overall, these results indicated that DNAm patterns form an independent group of data, quite distinct from the available phenotype, genotype or clinical attributes.

Because as many as 23 samples had missing information on the recurrence event (the largest number of missing values among data attributes), we also repeated factor analysis for all attributes except the recurrence status. In this case the data set contained a larger set of 75 samples with no missing values (29 CPC, 24 CPP, 22 aCPP). The general pattern of factor loadings was similar to the previously observed one (Additional file 2: Figure S8): in this case the top 3 factors were associated exclusively with DNAm patterns; Factor 4 was strongly associated with age and Factor 6 with death status and P53 mutation status, respectively. All other factors exclusively represented minor DNAm variations. As before, the overall results indicate that DNAm patterns form a distinct group of observations.
